# Primary Breast Lymphoplasmacytic Lymphoma in a Young Woman: A Rare Case of Waldenström’s Disease

**DOI:** 10.7759/cureus.97361

**Published:** 2025-11-20

**Authors:** Khaoula Amar, Fatima Zahra Bouabdellaoui, Mohammed El Magroud, Hafsa Taheri, Hanane Saadi, Amal Bennani, َAhmed Mimouni

**Affiliations:** 1 Gynecology, Mohammed VI University Hospital Center, Oujda, MAR; 2 Pathology, Mohammed VI University Hospital Center, Oujda, MAR; 3 Obstetrics and Gynecology, Mohammed VI University Hospital Center, Oujda, MAR

**Keywords:** breast, breast lymphoma, case report, lymphoplasmacytic, waldenström's macroglobulinemia

## Abstract

Primary breast lymphomas (PBLs) are rare tumors that originate in the breast without systemic disease at diagnosis. Lymphoplasmacytic lymphoma (LPL), usually associated with Waldenström’s macroglobulinemia, is exceptionally uncommon in this location. We report a case of a 36-year-old woman with no significant medical history who presented with a rapidly enlarging right breast mass. Imaging revealed a suspicious right breast lesion classified as Breast Imaging Reporting and Data System (BI-RADS) Category 4. Core needle biopsy with histopathology and immunohistochemistry confirmed CD20-negative LPL, an indolent B-cell lymphoma. The patient was treated with bendamustine-based chemotherapy and corticosteroids, with marked clinical and radiological improvement. This case emphasizes the importance of considering hematologic malignancies in the differential diagnosis of breast lesions to avoid unnecessary surgical management and ensure appropriate systemic therapy.

## Introduction

Most breast tumors are of epithelial origin, predominantly carcinomas. Primary breast lymphomas (PBLs) are rare entities, accounting for less than 0.5% of all breast malignancies and approximately 1-2% of extranodal non-Hodgkin lymphomas [1,2]. Among them, diffuse large B-cell lymphoma (DLBCL) and marginal zone lymphoma are the most common histological subtypes [3]. In contrast, lymphoplasmacytic lymphoma (LPL) - typically associated with Waldenström’s macroglobulinemia - is exceptionally uncommon in the breast, with only a few cases reported to date [4].

Recent studies have underscored the diagnostic challenges of PBL, particularly in rare subtypes, as clinical and radiological features often mimic those of primary breast carcinoma [1,5]. Misdiagnosis may result in delayed recognition or unnecessary surgical procedures [6]. Advances in histopathology, immunophenotyping, and, more recently, molecular testing have improved diagnostic accuracy, yet unusual presentations continue to pose significant challenges in daily practice [7,8].

Here, we report an unusual case of a CD20-negative primary breast LPL in a young woman. This case emphasizes the importance of considering hematologic malignancies in the differential diagnosis of breast tumors and highlights the need for tailored diagnostic and therapeutic strategies, including molecular testing such as MYD88 mutation analysis, in rare and atypical scenarios [4,9-11].

## Case presentation

A 36-year-old woman (gravida 2, para 2 (G2P2)), with no family history of breast cancer, presented with a rapidly enlarging right breast mass, self-detected four months prior. The lesion was associated with mild localized tenderness but no skin changes, nipple discharge, or systemic symptoms such as fever or weight loss. Her surgical history included two previous right breast lumpectomies. The first, performed four years earlier, reportedly revealed a benign lesion, though histological documentation was unavailable. The second, conducted four months prior at another institution, concluded to nonspecific chronic mastitis without atypia. Immunohistochemistry was negative for estrogen receptors (ER), E-cadherin, and epithelial membrane antigen (EMA). No further investigations were undertaken.

**Figure 1:**
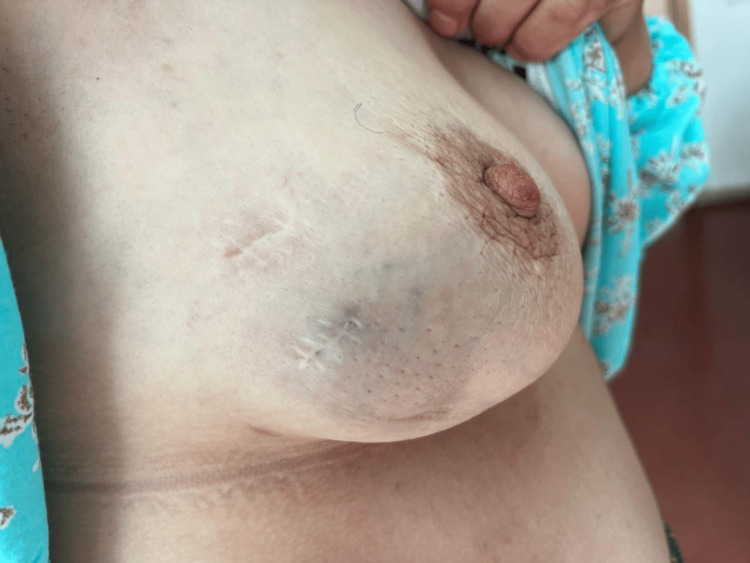
Clinical aspect of the right breast A palpable, well-circumscribed, rounded lesion was located in the lower inner quadrant of the right breast. The absence of skin changes, nipple retraction, or inflammatory signs was more consistent with a lymphoid rather than an epithelial breast malignancy.

On examination, the patient was in good general health. The right breast harbored a 4 × 3 cm firm, well-defined mass in the lower inner quadrant, adherent to superficial planes but mobile over deeper structures. A 2 cm mobile right axillary lymph node was also palpable (Figure 1). The left breast was normal.

**Figure 2:**
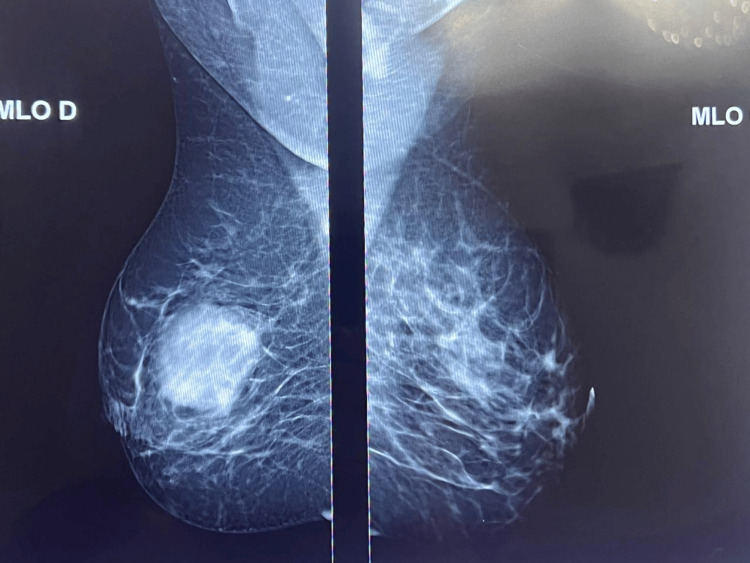
Mammographic appearance of the right breast A mediolateral oblique mammogram showed a solitary, well-defined, rounded opacity in the lower inner quadrant of the right breast. The lesion lacked spiculated margins or suspicious microcalcifications - findings that were atypical for carcinoma and may suggest a lymphoproliferative process.

Mammography revealed dense breasts (Breast Imaging Reporting and Data System (BI-RADS) Category 2) without significant abnormalities [12]. A fairly regular opacity was noted in the upper outer quadrant of the left breast, without microcalcifications, and was not suggestive of malignancy (Figure 2).

**Figure 3:**
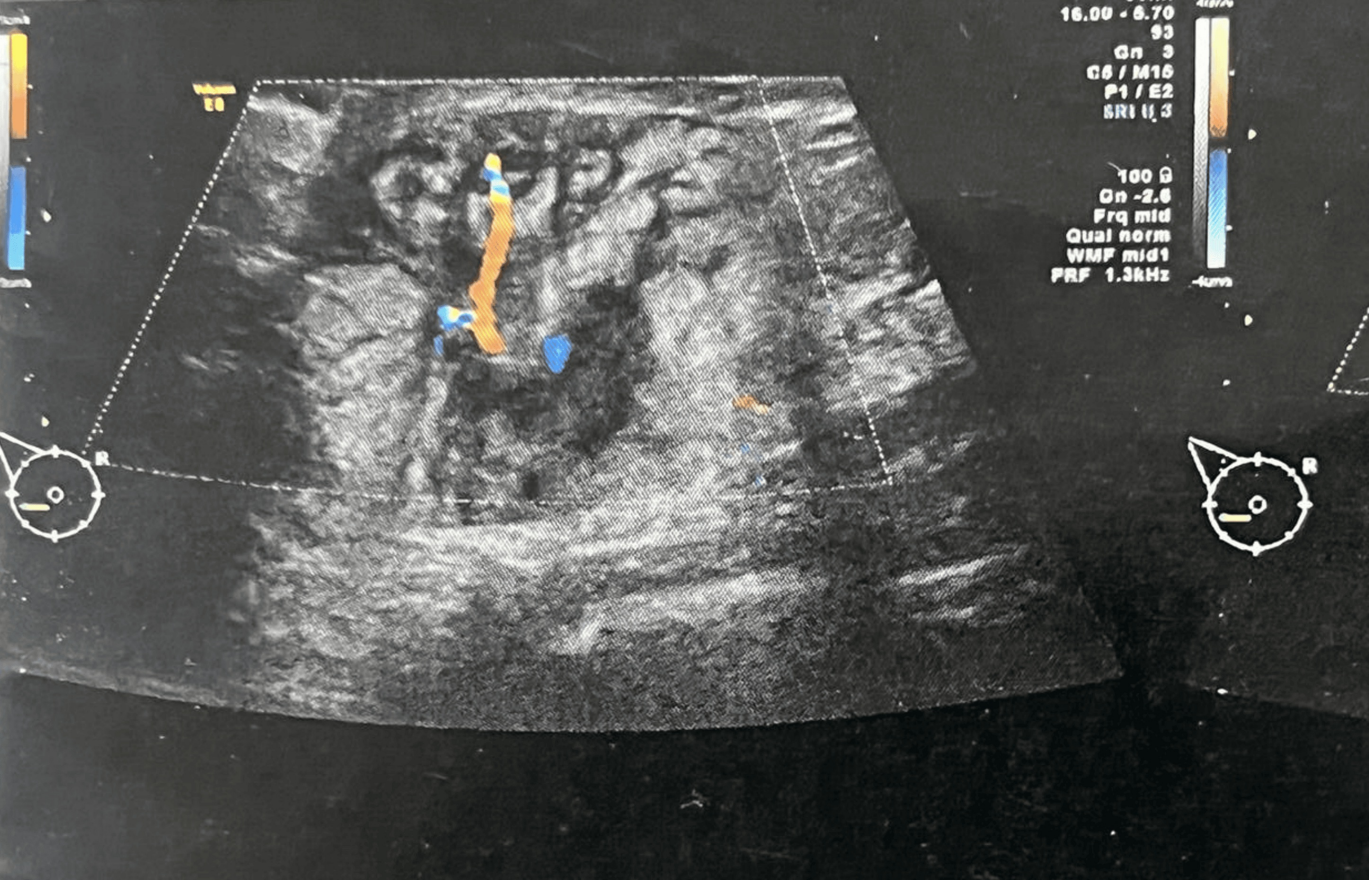
Ultrasound of the right breast Ultrasound imaging demonstrated a hypoechoic, heterogeneous, and hypervascular lesion with irregular margins measuring 3.7 × 2.7 cm. The marked vascularity and absence of posterior acoustic shadowing were imaging features supportive of lymphoma.

Ultrasound of the right breast revealed a hypoechoic, heterogeneous lesion with irregular margins and marked hypervascularization in the lower inner quadrant, measuring 3.7 × 2.7 cm (Figure 3), classified as BI-RADS Category 4 and associated with a 15 mm right axillary lymphadenopathy [12].

**Figure 4:**
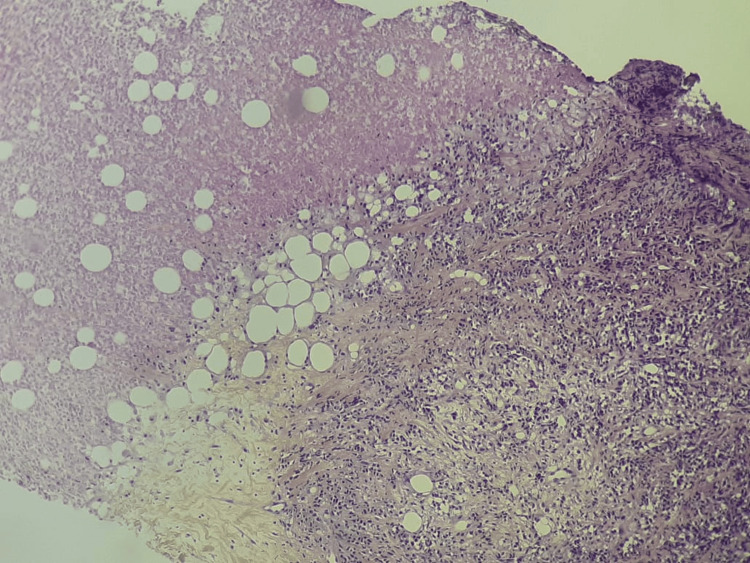
Histopathological features (H&E, ×100) Diffuse sheet-like proliferation of small lymphoid tumor cells with evidence of tumoral necrosis was observed.

**Figure 5:**
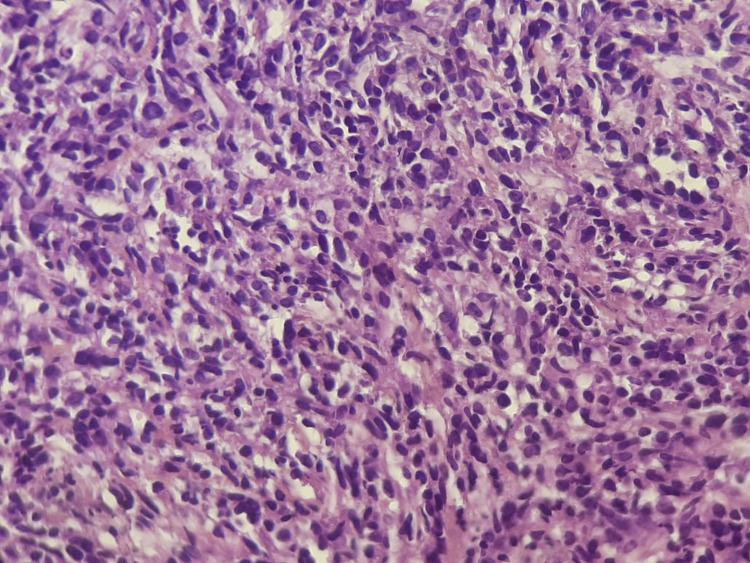
Histopathological features (H&E, ×40) At higher magnification, the tumor cells displayed abundant eosinophilic cytoplasm, round nuclei with irregular contours, dense chromatin, and occasional visible nucleoli. Focal areas of plasmacytic differentiation were also observed.

A core needle biopsy was performed. Histological analysis showed a diffuse solid proliferation of small to medium-sized lymphoid cells with eosinophilic cytoplasm and large, round or ovoid, hyperchromatic nuclei, featuring occasional prominent nucleoli and focal plasmacytoid differentiation. The tumor proliferation index (Ki-67) was estimated at 30% (Figures 4-5).

**Figure 6:**
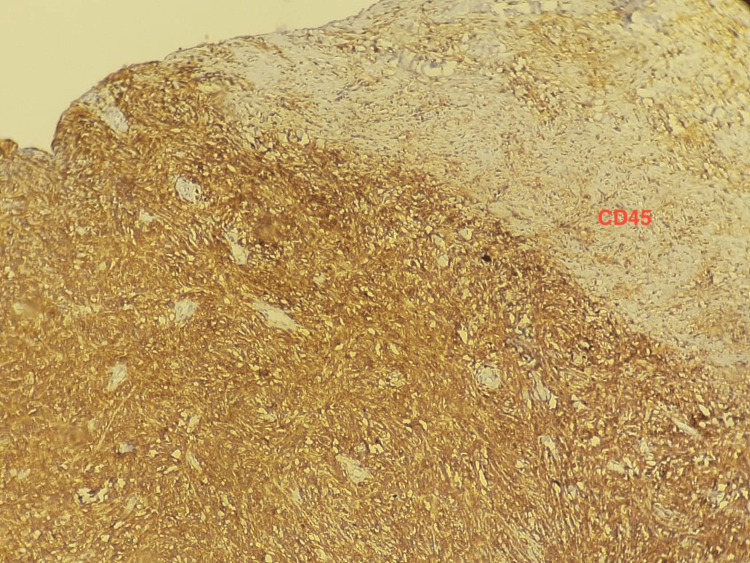
Immunohistochemical staining for CD45 Immunohistochemical analysis at high magnification revealed diffuse and strong cellular positivity for CD45.

**Figure 7:**
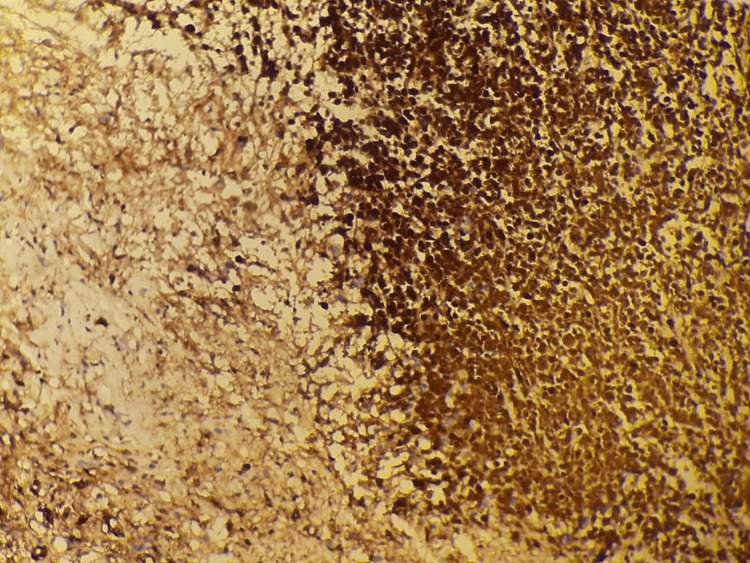
Immunohistochemical staining for IgM Immunohistochemical analysis at high magnification revealed diffuse and strong cellular positivity for IgM.

Immunohistochemistry was positive for CD45, BCL2, IgM, CD68, S100, CD79a (heterogeneous), CD138 (focal), and GATA3 (focal). Markers for CD20, CD3, ER, PR, PAX5, and pan-cytokeratin were negative. The profile was compatible with LPL of the breast (Figures 6-7).

A staging workup with bone marrow biopsy and thoraco-abdominopelvic CT scan showed no bone marrow infiltration or other extranodal involvement, fulfilling Wiseman and Liao’s criteria for PBL [13].

The patient was started on bendamustine combined with corticosteroids. After the fourth cycle, significant clinical and radiological improvement was observed, with a >50% reduction in tumor size and complete resolution of axillary lymphadenopathy. She remains in good condition on follow-up.

## Discussion

PBL is a rare clinical entity, representing less than 0.5% of all malignant breast tumors and approximately 1-2% of all extranodal lymphomas [1,14]. This pathology generally affects women, although cases in men have been reported. Regarding age, two peaks of frequency have been noted: a first peak in young women of reproductive age and a second peak between 50 and 60 years. The two most frequently reported histological subtypes are DLBCL and marginal zone lymphoma [3]. LPL, usually associated with Waldenström’s macroglobulinemia, is exceptionally rare in this location, with only a few isolated cases published in the literature [4,15].

The clinical presentation of PBL is often nonspecific, typically manifesting as a unilateral, painless, palpable mass, sometimes associated with axillary lymphadenopathy but rarely with systemic B symptoms (fever, night sweats, weight loss) [14]. In our case, the patient was a 36-year-old woman presenting with a rapidly enlarging, firm mass with ipsilateral mobile lymphadenopathy in the absence of inflammatory signs. This presentation closely mimicked breast carcinoma, which is consistent with the diagnostic pitfalls described in the literature [1,6].

Additionally, imaging findings are nonspecific. Mammography often shows well-defined, homogeneous, noncalcified masses without architectural distortion, with a benign appearance, mimicking a cyst, fibroadenoma, or phyllodes tumor. Less frequently, it may resemble mastitis with diffuse breast density increase, a poorly defined mass, or a spiculated mass [6]. On ultrasound, PBL most commonly presents as a homogeneous, hypoechoic mass with well-circumscribed margins; more rarely, as a heterogeneous, hypoechoic lesion resembling mastitis [2,16]. MRI, although not performed in this case, may show nonspecific enhancements and can be useful for local staging [17]. However, imaging alone cannot reliably distinguish lymphoma from carcinoma, and tissue sampling remains mandatory.

Diagnosis is histological, obtained after core needle or surgical biopsy. Frozen section carries a high risk of error, as the differential diagnosis may include anaplastic or medullary carcinoma. Immunohistochemical analysis with lymphoid markers is essential for confirmation. LPL is characterized by small lymphoid cells with plasmacytoid differentiation, a low to intermediate Ki-67 index, and the expression of B-cell markers such as CD20, CD79a, and surface IgM [18]. The immunohistochemical profile of our case is summarized in Table 1.

**Table 1:** Immunohistochemistry profile of the breast lesion

Marker	Result	Interpretation
CD45	Positive (diffuse, strong)	Confirms lymphoid origin of tumor cells
CD20	Negative	Rare finding in lymphoplasmacytic lymphoma; excludes anti-CD20 therapy
CD79a	Positive	Supports B-cell lineage
IgM	Positive (cytoplasmic)	Indicates plasmacytic differentiation

The tumor cells in our case showed no CD20 expression, a rare but documented finding, which may complicate diagnosis and reflects biological heterogeneity [19]. The absence of CD20 has major therapeutic implications, as it precludes the use of rituximab, the cornerstone of most B-cell lymphoma treatments. Consequently, alternative chemotherapy regimens must be considered. Although molecular testing for the MYD88 L265P mutation - strongly associated with Waldenström’s macroglobulinemia - was not available in our setting, its detection could further refine the diagnosis and guide the use of targeted therapies [10].

Staging is essential to determine whether the breast involvement is primary or secondary to systemic lymphoma. According to Wiseman and Liao’s criteria [13], the diagnosis of PBL requires adequate pathological specimens, a close association between breast and lymphomatous tissue, absence of disseminated disease at diagnosis, and no history of prior lymphoma. Our patient underwent a bone marrow biopsy and thoraco-abdominal CT scan, both of which were normal. These findings confirmed the diagnosis of primary breast involvement rather than secondary dissemination.

The management of primary breast LPL mirrors that of lymphomas in other extranodal locations. Multiple protocols have been reported in the literature. Currently, most authors recommend chemotherapy based on cyclophosphamide, vincristine, and prednisone, sometimes combined with anti-CD20 immunotherapy. In CD20-negative lymphomas, the lack of a rituximab target necessitates regimens based on alkylating agents such as bendamustine or chlorambucil [20]. Novel molecules, particularly BTK inhibitors, represent a promising option for selected patients, especially those harboring MYD88 mutations [11,21]. Radiotherapy may be considered in localized forms, though its role remains debated. Surgery, whether lumpectomy or mastectomy, has no therapeutic indication and should only be performed for diagnostic purposes [3,6].

This case has several distinctive features: the young age of the patient, the absence of systemic symptoms, the lack of dissemination, and the CD20-negative immunophenotype. Compared with previously reported cases, this presentation remains exceptionally rare. It highlights the importance of considering malignant hematologic disorders in the differential diagnosis of atypical breast masses, underscores the role of immunohistochemistry and molecular testing in guiding therapy, and stresses the need for individualized treatment strategies. Early referral to hematology-oncology is essential for optimizing outcomes.

## Conclusions

CD20-negative LPL of the breast is an exceptional entity that can mimic carcinoma. Accurate diagnosis relies on immunohistochemistry, and management requires timely systemic therapy. Molecular testing and further case reports are needed to optimize future diagnostic and therapeutic strategies.
